# Inadvertent filtering bleb due to extracapsular cataract extraction wound reopening after mitomycin C use: a case report

**DOI:** 10.1186/s13256-023-03784-6

**Published:** 2023-02-18

**Authors:** Phit Upaphong, Kessara Pathanapitoon, Winai Chaidaroon

**Affiliations:** Department of Ophthalmology, Faculty of Medicine, Chiang Mai University, 110 Intawaroros Road, Chiang Mai, 50200 Thailand

**Keywords:** Conjunctival bleb, Long-term adverse event, MMC, Pterygium excision, Wound leakage

## Abstract

**Background:**

Mitomycin C has been used adjunctively in various procedures, including pterygium excision. Delayed wound healing, the long-term complication of mitomycin C, can occur several years later and may rarely cause a subsequent inadvertent filtering bleb. However, conjunctival bleb formation from the reopening of an adjacent surgical wound after mitomycin C use has not been reported.

**Case presentation:**

A 91-year-old Thai woman had undergone pterygium excision 26 years ago, with adjunctive mitomycin C, as well as an uneventful extracapsular cataract extraction in the same year. The patient developed a filtering bleb without glaucoma surgery or trauma approximately 25 years later. Anterior segment ocular coherence tomography illustrated a fistula connected between the bleb and anterior chamber at the scleral spur. The bleb was observed without further management, as no hypotony or bleb-related complications occurred. The symptoms/signs of bleb-related infection were advised.

**Conclusions:**

This is a case report of a rare novel complication of mitomycin C application. Conjunctival bleb formation from the reopening of surgical wound, which was related to the previous mitomycin C use, could occur after a few decades.

## Background

Mitomycin C (MMC) is an alkylating agent that is used in ophthalmology as an anti-fibrotic drug [1]. MMC minimizes scar formation via the inhibition of keratocyte activation and myofibroblast differentiation [1]. Due to these effects, topical MMC has been used adjunctively in various procedures, including pterygium excision, to decrease the recurrence rate of pterygium, and for trabeculectomy to maintain the aqueous outflow and improve the efficacy of intraocular pressure (IOP) reduction [1, 2]. However, the contact of MMC with limbal stem cells and with fibroblasts in scleral tissue could inhibit proliferation of these cells [1, 2]. Hence, MMC is associated with several complications, for instance, delayed wound healing, scleral melting, thin atrophic blebs, and leaking blebs [1, 2]. These sequelae can occur months to several years after surgery [2–6].

Herein, we present a rare case with an inadvertent bleb formation in the eye, more than 20 years after pterygium excision with an adjunctive MMC and extracapsular cataract extraction (ECCE).

## Case presentation

**Fig. 1:**
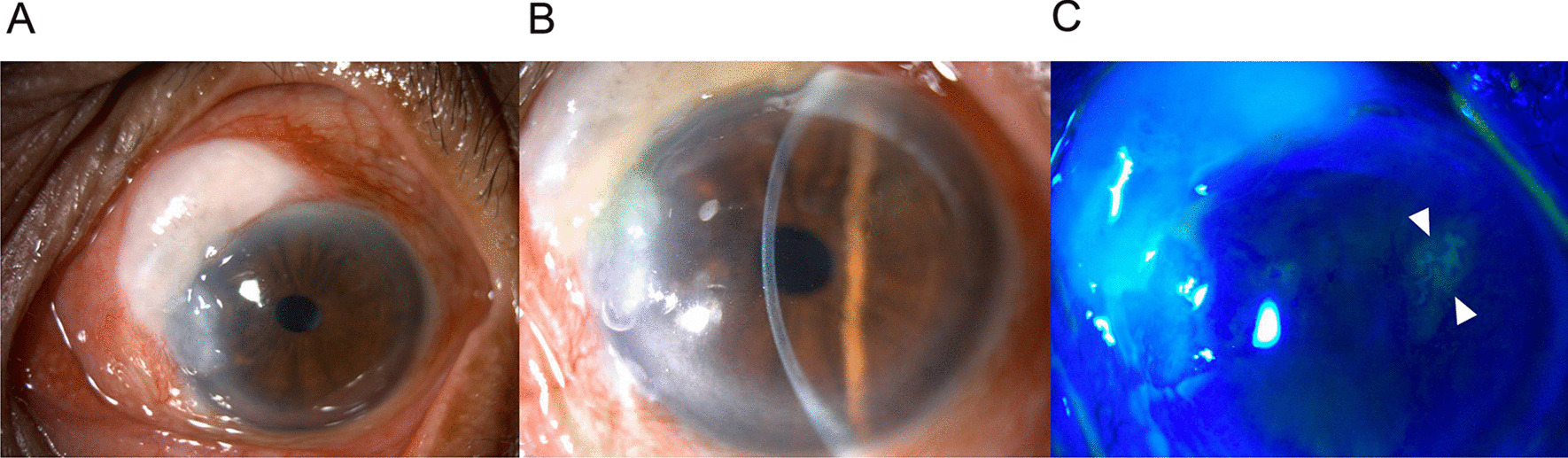
Composite slit-lamp images of the left eye. **a** A localized encapsulated bleb with scar tissue in a ring of steel configuration on the superonasal conjunctiva at ten times magnification. Adjacent to the inferior part of the bleb, just above the site of the previous pterygium, is a nodular corneal degeneration and corneal scar. The diffuse conjunctival injection is also observed. **b** Deep anterior chamber and a sign of epitheliopathy (arrowheads) observed at 16 times magnification. **c** An examination with fluorescein staining was observed using blue light. The stain highlights the area of the dendritic ulcer and terminal bulbs (arrowheads). The Seidel test was negative over the bleb

A 91-year-old Thai woman with bilateral pseudophakia presented with acute left eye pain and redness. The best-corrected visual acuity was 6/9. On slit-lamp examination of the left eye, diffuse conjunctiva injection (Fig. 1a) and the dendritic ulcer with terminal bulbs, highlighted by fluorescein staining, were observed on the superotemporal side of the cornea (Fig. 1b, c). There were no cells in the anterior chamber. She was diagnosed with herpes simplex keratitis (HSK) on the left eye. Noticeably, there was also a localized encapsulated bleb on the superonasal conjunctiva with a negative Seidel test (Fig. 1a, c). No peripheral iridectomy was seen and the IOP was 12 mmHg. Fundus examination revealed a cup-to-disc ratio of 0.5 with an intact neural rim.

The patient had no underlying systemic/ocular diseases, or any significant trauma to this eye, and denied use of any topical anti-glaucoma medications. According to the medical record, she had only had two surgeries; the first operation was a nasal pterygium excision with bare sclera technique and adjunctive MMC performed 26 years ago. Unfortunately, the operative note of the previous pterygium excision, including the concentration and duration of MMC, was unavailable. The second operation was an uneventful ECCE with intraocular lens implantation 8 months after the pterygium excision. She was seen for 3 years at our hospital without any complications and then was lost to follow-up. The first notice of the bleb was approximately a year ago, which was 25 years after the surgeries.

**Fig. 2:**
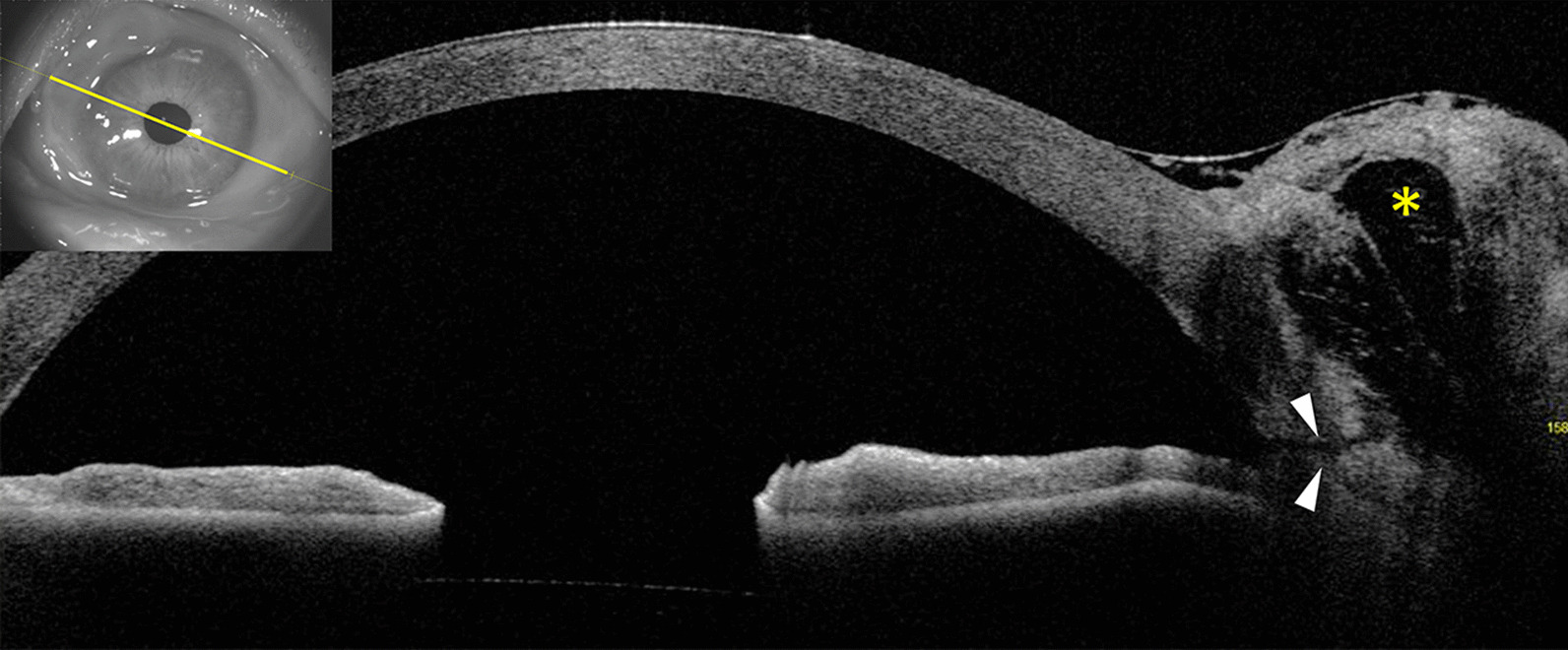
An image from the anterior segment ocular coherence tomography. The asterisk indicates a thick-wall cystic space. A straight and sharp-edged fistula parallel to the iris plane, which connects between the bleb and anterior chamber at the scleral spur, is observed (arrowheads)

Gonioscopic examination revealed 5° of a localized area of peripheral anterior synechiae (PAS) at the superonasal quadrant anterior chamber angle without any fistula or fish-mouthing appearance of wound gape. Anterior segment ocular coherence tomography (ASOCT) illustrated a thick-wall cystic space (Fig. 2). Noticeably, at the 158° cut, a straight, sharp-edged fistula, parallel to the iris plane and connected between the bleb and anterior chamber at the scleral spur, was also revealed (Fig. 2). These characteristics corresponded with the site of the ECCE scleral wound. The location of the fistula from ASOCT coincided with the PAS in the gonioscopic view. According to the area of pterygium scar, it is highly suggestive that the site of this fistula was within the area of previous MMC application. An inadvertent bleb formation, the late complication of MMC, was diagnosed.

She was treated successfully with a 1 week course of hospital-made topical 2% ganciclovir, five times daily for herpes simplex keratitis. The bleb was observed without further management as it was inactive and had no ocular hypotony. The patient had been informed about the warning symptoms/signs of bleb-related infection.

## Discussion and conclusions

This is a case of an inadvertent bleb after pterygium excision, with an adjunctive MMC and ECCE. According to the history of MMC use and findings from ASOCT, this was a highly probable late complication of MMC [7]. In glaucoma filtration surgery, such as trabeculectomy, complications of MMC are likely to be associated with its concentration [8]. The inadvertent bleb formation related to previous MMC use is rare. To the best of our knowledge, only one publication [5] reported this complication, 4 years after using MMC in pterygium excision. The fistula in that publication [5] was located at the thin scleral bed at the site of pterygium excision. In contrast, the fistula in our publication was located at the scleral spur, which coincided with the previous ECCE scleral incision adjacent to the area of previous pterygium surgery. According to the configurations of the fistula from ASOCT, it is suggested that MMC may cause a reopening of the previous surgical wound after 20 years of administration. Having both pros and cons, MMC is still under consideration for whether it should be used in primary pterygium [2].

An inadvertent bleb after uneventful cataract surgery, without an association with MMC, is also rare [4]. There have been only a few reports of these complications ranging from 2 years to over 10 years after cataract extractions [4, 6]. The underlying cause was from internal wound gape observed from gonioscopy [6]. From the literature, scleral incisions were more susceptible to the internal wound gape, leading to wound instability compared with a clear corneal incision [9]. So, it is less likely to happen following a phacoemulsification technique [6].

In terms of treatment, as the fibrotic bleb, in this case, was nonfunctional and there was no bleb-related complication, excision of the bleb may not be required [4]. However, late bleb leaks in this patient are still possible due to the complication of MMC [2–6]. Bindlish *et al.* reported 14.6% of bleb leaks occurred at a mean of 27.9 months [3]. Hence, surveillance for further leakage and subsequent blebitis or endophthalmitis is crucial [4]. Excision of the bleb is indicated when the wall is thin and avascular, due to the higher chance of leakage [4]. Despite no wound gape seen on the gonioscopy, Krishnacharya *et al.* found that fluid aspiration of the bleb is not successful as there still be a small connection between the bleb and anterior chamber leading to a recurrence [6]. In case of sight-threatening sequelae from an overfiltration of aqueous humor such as hypotony, maculopathy/choroidal detachment, or shallow anterior chamber, bleb excision with defect closure is considered [4–6].

The strength of this case report is that the follow-up duration after both surgeries was sufficient to exclude any early post-operative complications. Furthermore, ASOCT imaging was performed to illustrate the underlying pathology. However, the limitations include (1) no availability of concentration and duration of MMC used in the previous pterygium excision, and (2) no ophthalmologist appointment at the onset of bleb formation.

In conclusion, this is a rare case report of a late complication of MMC causing reopening of the previous ECCE scleral wound—an inadvertent bleb. Without the characteristics of a bleb prone to leakage and the bleb-related complications, it is reasonable to just observe and follow up. Nonetheless, this report was not meant to discourage ophthalmologists from using this medication.

## Data Availability

The datasets used and/or analyzed during the current study are available from the corresponding author on reasonable request. Data available on request from the authors.

## Abbreviations

ASOCT: Anterior segment ocular coherence tomography
ECCE: Extracapsular cataract extraction
HSK: Herpes simplex keratitis
IOP: Intraocular pressure
MMC: Mitomycin C
